# Decomposing Neural Synchrony: Toward an Explanation for Near-Zero Phase-Lag in Cortical Oscillatory Networks

**DOI:** 10.1371/journal.pone.0003649

**Published:** 2008-11-06

**Authors:** Rajasimhan Rajagovindan, Mingzhou Ding

**Affiliations:** The J. Crayton Pruitt Family Department of Biomedical Engineering, University of Florida, Gainesville, Florida, United States of America; Indiana University, United States of America

## Abstract

**Background:**

Synchronized oscillation in cortical networks has been suggested as a mechanism for diverse functions ranging from perceptual binding to memory formation to sensorimotor integration. Concomitant with synchronization is the occurrence of near-zero phase-lag often observed between network components. Recent theories have considered the importance of this phenomenon in establishing an effective communication framework among neuronal ensembles.

**Methodology/Principal Findings:**

Two factors, among possibly others, can be hypothesized to contribute to the near-zero phase-lag relationship: (1) positively correlated common input with no significant relative time delay and (2) bidirectional interaction. Thus far, no empirical test of these hypotheses has been possible for lack of means to tease apart the specific causes underlying the observed synchrony. In this work simulation examples were first used to illustrate the ideas. A quantitative method that decomposes the statistical interdependence between two cortical areas into a feed-forward, a feed-back and a common-input component was then introduced and applied to test the hypotheses on multichannel local field potential recordings from two behaving monkeys.

**Conclusion/Significance:**

The near-zero phase-lag phenomenon is important in the study of large-scale oscillatory networks. A rigorous mathematical theorem is used for the first time to empirically examine the factors that contribute to this phenomenon. Given the critical role that oscillatory activity is likely to play in the regulation of biological processes at all levels, the significance of the proposed method may extend beyond systems neuroscience, the level at which the present analysis is conceived and performed.

## Introduction

Cortical information processing involves the coordinated activity among many distinct regions of the brain. Statistically, this coordinated activity manifests as correlated or synchronized co-variations in the recorded multivariate data. Early studies in animal preparations have shown that stimulus-evoked short-range synchrony between neurons in the primary visual area subserves perceptual binding of sensory information [1], [2]. Simultaneous action potentials fired by lower order neurons [3]–[5] provide an effective drive on higher order neurons, the activations of which enable object discrimination and perception. In humans, similar observations have been made [6] where highly synchronized EEG activity occurs in response to stimulus input. In all these cases, a near-zero phase-lag relation between different data series is observed, reflecting the millisecond or even sub-millisecond precision required for feature integration [1], [7]–[9].

Increasingly, long-range synchronization of oscillatory field activity with near-zero phase-lag, often in the absence of stimulus input, has been reported [8], [10]–[13]. Depending on the task, the strength of the synchronization can influence the efficacy of both sensory and motor processing [12], [14]–[16], suggesting that it has a functional role. To date, however, an explanation of the observed near-zero phase-lag relation in these large-scale networks has not been forthcoming. Recent work has considered the importance of the near-zero phase-lag phenomenon from the perspective of neuronal communication. In particular, field oscillations provide an index of the excitability level of a neuronal ensemble [17], [18]. During the excitable phase of the oscillation cycle, presynaptic neurons are more likely to fire action potentials, whereas for the postsynaptic neuron, action potentials received during the excitable phase are more effectively integrated, leading to a response [18]–[20]. This suggests that long-range synchrony could serve as a gating mechanism of information flow in cortical circuits [20]–[23]. Given that the conduction delay between two brain areas is only a small fraction of the oscillation cycle, a near-zero phase-lag relation could stem either from reciprocal communication between the two areas (bidirectional interaction) or from the two areas being readied to communicate by a third set of areas (common input). Mathematically, it is intuitively clear that a positively correlated common input with no significant relative time delay, if strong enough, can drive the two areas into near-zero phase-lag synchrony. Alternatively, a recent computational model based on the anatomical connectivity pattern in the visual system examined the sufficient conditions underlying the emergence of near-zero phase-lag synchrony in cortical networks experiencing bidirectional interaction [24]. The testing of these possibilities has not been carried out empirically. The main reason is that the commonly used methods such as cross correlation and coherence lack the ability to decompose neural interactions into their constituent components.

In this paper we attempt to address this problem by introducing Geweke's time series decomposition theorem into the analysis of multivariate neural data. Let the two brain areas be denoted by A and B. The interaction between these two areas may be mediated by A influencing B (A→B), B influencing A (B→A), and/or A and B both receiving a common input. Geweke's theorem states that the total interdependence (synchrony) between two stochastic processes from A and B can be expressed as the sum of the three components: (A→B)+(B→A)+(instantaneously correlated common input). Here the arrow is understood in the sense of Granger causality and the instantaneously correlated common input is represented as instantaneous causality [25]–[28]. In this framework it is hypothesized that bidirectional interaction or positively correlated common input or a combination of both can contribute to the establishment of near-zero phase-lag synchrony. In particular, when the interaction is clearly unidirectional (e.g. A→B equals zero but B→A does not), the phenomenon of near-zero phase-lag is likely to be the result of strong positively correlated common input with no significant relative time delay, arising exogenously to A and B. We tested these ideas by first using simulation examples and then analyzing local field potential data recorded from behaving monkeys performing a visuomotor pattern discrimination task.

## Materials and Methods

### Simulation

#### Setup

Auto-regressive model of the form in Eq. (1) was used to generate all the simulated time series. Two representative types of interaction pattern, namely (a) unidirectional interaction with positively correlated common input and (b) bidirectional interaction, were considered. The phase-lag as a function of appropriate model parameters for both cases were studied.

#### Positively correlated common input

A bivariate AR(3) process [p = 3 in Eq. (1)] in which X drives Y was used. The coefficients of the model were a_1_ = 0.4428, a_2_ = −0.5134, a_3_ = 0, d_1_ = 0.506, d_2_ = −0.6703, d_3_ = 0, b_1_ = b_2_ = b_3_ = 0, c_1_ = c_2_ = 0, c_3_ = 0.1, and Σ_xx_ = Σ_yy_ = 1. The cross terms in the noise covariance matrix, Σ_yx_ = Σ_xy_, reflecting the strength of positively correlated common input, was systematically varied. The parameter choice above enabled the model to oscillate at 40 Hz for which the phase-lag was computed. The dataset consisted of 100 epochs of 200 sample points each. The sampling rate was assumed to be 200 Hz. Note that, for the given sampling rate, the input correlation can be considered contemporaneous or instantaneous since the noise terms in the AR model in Eq. (1) are not correlated over time.

#### Bidirectional interaction

A bivariate AR(4) process [p = 4 in Eq. (1)] was used. The coefficients of the model were a_1_ = 0.9, a_2_ = −0.5, a_3_ = 0, a_4_ = 0, d_1_ = 0.8, d_2_ = −0.5, d_3_ = d_4_ = 0, Σ_xx_ = Σ_yy_ = 1, and Σ_xy_ = Σ_yx_ = 0 (no common input). The coefficients of the interaction terms b_1,2,3,4_ and c_1,2,3,4_ were varied in tandem to achieve simultaneous increase in the strength of both feed-forward and feed-back interaction. The model exhibited narrow frequency band oscillations with a frequency peak at 32 Hz for which the phase-lag was computed. The dataset consisted of 100 epochs of 200 sample points each. The sampling rate was assumed to be 200 Hz.

### Experiment

#### Behavioral paradigm

Two monkeys (GE and LU) were trained to perform a GO/NO-GO visual pattern discrimination task in the Laboratory of Neuropsychology at the National Institute of Mental Health [10], [29]. Animal care was in accordance with institutional guidelines at the time. The monkey initiated each trial by depressing a hand lever and maintained its depression while anticipating the onset of a visual stimulus. Four squares arranged in either a line (left-slanting and right-slanting) or a diamond (left-slanting and right-slanting) shaped formation appeared on a visual display after a random time interval triggered by the lever depression. The monkey made either a GO (lever release) or a NO-GO (maintaining lever depression) response upon discriminating the input pattern. For the GO trials, the time between stimulus onset and the lever release is defined as the response time (RT). The experiment was conducted in sessions of approximately 1000 trials each.

#### Data acquisition

Local field potentials (LFPs) were recorded with bipolar teflon-coated platinum microelectrodes (51-µm diameter and 2.5-mm tip separation) from up to 15 distributed sites located in the hemisphere contralateral to the dominant hand (right hemisphere in monkey GE and left hemisphere in monkey LU). The data collection period started 90 ms before the stimulus onset and ended approximately 500 ms after stimulus onset [10]. LFPs were amplified by Grass P511J amplifiers (−6 dB at 1 and 100 Hz, 6 dB/octave falloff) and digitized at 200 Hz. As this study is concerned with the phase-lag between two signals, the bipolar derivation carries certain arbitrariness. Reversing the order of the two subtracting electrode leads can change the phase from 0 to π or vice versa. This can affect the formulation of the hypothesis to be tested. See Results and Discussion sections for more details.

#### Data set selection

Previous analysis of the same experiment [11], [30] has identified a coherent beta (14 to 30 Hz) oscillatory network in the sensorimotor cortex involving both pre- and post-central sites during the prestimulus time period. For this work the three recording sites that are common to both monkeys were selected for further analysis: primary somatosensory area (S1), primary motor area (M1) area, and posterior parietal area 7b. Trials contaminated with artifacts or associated with incorrect behavioral responses were rejected. To achieve a sufficient number of trials, different sessions having similar RT distributions were combined to yield a data set of approximately 2400 and 1400 trials for monkeys GE and LU, respectively. The time interval from −90 ms to 20 ms was considered, which was 110 ms in duration and contained 22 sample points. We henceforth refer to this time interval the prestimulus time interval since it took the stimulus more than 20 ms to reach the cortex.

#### Time series decomposition

Let the LFP data from two recording sites be denoted by *X*_t_ and *Y*_t_. Jointly, they can be represented by the following bivariate autoregressive model
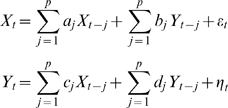
(1)where the noise terms *ε_t_* and *η_t_* are uncorrelated over time, and their contemporaneous covariance matrix is
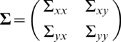
(2)If *b_j_* is not uniformly zero for *j* = 1,2,…, *p*, then *Y*_t_ is said to have a causal influence on *X*_t_. Likewise, *X*_t_ is said to have a causal influence on *Y*_t_ if *c_j_* is not uniformly zero for *j* = 1,2,…, *p*. If Σ*_xy_* = Σ*_yx_*≠0, indicating that the noise terms *ε_t_* and *η_t_* are correlated instantaneously, then the interdependence between *X*_t_ and *Y*_t_ has another contributor that is not explained by the interaction between *X*_t_ and *Y*_t_. This contributor, possibly representing influences exogenous to the (X,Y) system such as a common input with no significant relative time delay from a third system, will be referred to as the instantaneous causality [26], [27].

Fourier transforming Eq. (1) and performing proper ensemble average, we obtain the spectral matrix
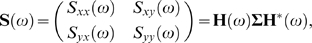
(3)where * denotes complex conjugate and matrix transpose, and
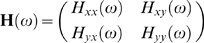
(4)is the transfer function matrix. The total interdependence between *X*_t_ and *Y*_t_ at frequency *f* (ω = 2πf) is defined as
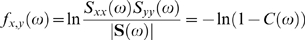
(5)where 
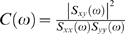
 is the coherence function. The phase-lag between *X*_t_ and *Y*_t_ at a given frequency is given by 
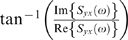
. The Granger causality between the two time series is
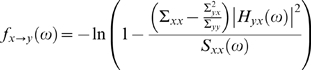
(6)and
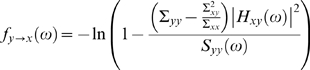
(7)In addition, the frequency domain expression for the instantaneous causality is
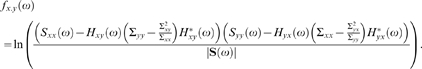
(8)


It can be shown that the above set of variables are related through
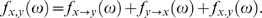
(9)Intuitively, the decomposition in Eq. (9) means that the total amount of statistical synchrony between two LFP signals is the sum of their causal drives on one another and a common input component. The expression in Eq. (9) can be integrated over the entire frequency domain to yield the time-domain counterpart:
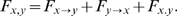
(10)


While the frequency-domain formulation [25] is convenient for estimation, the above time-domain decomposition is more readily interpretable and will be used here.

#### Data analysis protocol

For each monkey there are 3 distinct pairs of recording sites: (M1, S1), (M1, 7b) and (S1, 7b). For each pair, previous work [11], [30] has identified a prominent coherence peak in the beta frequency range (14 to 30 Hz). Except for (M1, S1) in monkey LU, the coherence in other five channel pairs is concentrated in the beta frequency range. These five channel pairs are further analyzed due to the reason that for these channel pairs, the time-domain quantities are equivalent to that in the beta range and as pointed out earlier, the Geweke decomposition is more readily interpretable in the time domain. The relative phase, referred to as the phase-lag in this study, is well-defined for the peak frequency. The dependence of this phase-lag on the factors in Eqs. (9) and (10) was investigated by carrying out the following procedure:

1. For a given pair of recording sites, the phase-lag in the beta frequency range was estimated for each single trial by Fast Fourier Transform (FFT) and sorted according to its value. The sorted trials were grouped into subensembles with 30% overlap, resulting in 33 and 19 groups in monkeys GE and LU, respectively. Each subensemble contained 100 trials.
2. For each subensemble the ensemble mean was estimated and removed from the individual trials within the subensemble. This is to ensure that the data may be treated as coming from a zero-mean stochastic process.
3. An AR model of order *p* = 9 was fit to the mean-removed data in each subensemble. Power, coherence, causality spectra as well as phase-lag at the peak frequency were derived from the AR model [25], [26].
4. Spearman rank correlation (SRCC) and Spearman rank partial correlation [31] (SRPCC) were computed between the instantaneous causality measure and the estimated phase-lag to assess the prediction that the two variables are negatively correlated. To remove the effect of directional influences, a partial correlation analysis was performed. Significance was determined through one tail t-test with a significance level of p<0.05.
5. For channel pairs determined to have bidirectional interactions, Spearman rank correlation (SRCC) and Spearman rank partial correlation [31] (SRPCC) were computed between the magnitude of the sum of the two directional influences and the estimated phase-lag to assess whether they exhibit negative correlation.

#### Logic of the analysis protocol

The goal of this work is to test that (a) positively correlated common input with no significant relative time delay and (b) bidirectional interaction contribute to the formation of near-zero phase-lag. In the simulation examples, this is accomplished by changing the strength of input correlation and bidirectional interaction and observing the corresponding change in the phase-lag. For actual data, while Geweke's theorem allows the extraction of various causal influences through the decomposition of synchrony, the strength of input correlation and bidirectional interaction is not easily manipulated. The sorting of trials according to their phase-lag is the strategy to deal with this problem. Each subensemble of trials gives rise to a different phase-lag value. Performing Geweke's decomposition for each subensemble provides the avenue to observe the correlation between phase-lag and common input/bidirectional interaction. It is important to note that, for a given pair of recording sites, both instantaneous causality and the two directional influences may change as functions of the sorted phase-lag. A simple pairwise correlation analysis may thus become confounded. Partial correlation is used here to make possible the examination of one factor's contribution to near-zero phase-lag with the contribution of other factors statistically removed. To generalize the above five-step protocol to other problems of interest, one can replace beta frequency by other relevant frequencies and suitably modify the subensemble size and the degree of overlap. In addition, since phase-lag is a bivariate phenomenon involving two simultaneous time series and our hypothesis does not distinguish whether the common input stems from the sites within the multivariate data set or sources not observed in the experiment, a pairwise analysis is sufficient for the purpose of this study. In general, however, one may wish to apply conditional Granger causality [25], [26] to ascertain that the causal influence between two recording sites is not mediated by other recorded sites before applying the above analysis protocol.

## Results

### Simulation

The impact of (a) instantaneously positively correlated common input and (b) directional interaction on phase-lag is examined using simulated time series and summarized in Tables 1 and 2. In the case of the AR(3) model with unidirectional interaction, increased correlation in common input, as measured by increased instantaneous causality, reduces the magnitude of phase-lag from 2.19 radians to a near-zero value of 0.16 radians (Table 1). Similar effect was observed in networks with bidirectional interaction (not shown). In the case of the AR(4) model, the parametric increase in the model coefficients b_1,2,3,4_ and c_1,2,3,4_ in Eq. (1) resulted in increased bidirectional interaction, as measured by increase in feed-forward and feed-back causal influences, leading to decrease in the magnitude of phase-lag to near-zero values (Table 2).

**Table 1:** Influence of increased instantaneous causality on phase-lag.

Network with unidirectional interaction pattern	
Instantaneous Causality	Phase-lag (radians)
0.00	2.19
0.04	1.16
0.29	0.44
1.02	0.16

Simulated data were generated by a bivariate AR(3) model with unidirectional interaction. Here instantaneous causality characterizes the strength of correlation in common input. Magnitude of phase-lag is seen to decrease with increase in instantaneous causality.

**Table 2:** Effect of increased bidirectional interaction on phase-lag.

Network with bidirectional interaction pattern		
F_X→Y_/F_Y→X_	F_X→Y_+F_Y→X_	Phase-lag (radians)
0.038/0.081	0.119	1.31
0.085/0.117	0.202	0.94
0.267/0.131	0.398	0.36

Simulated data were generated by a bivariate AR(4) model with bidirectional interaction. F_X→Y_ and F_Y→X_ denote the feed-forward and feed-back causal influences. Magnitude of phase-lag is seen to decrease with increase in strength of bidirectional interaction.

### Experiment

#### Network identification

**Figure 1:**
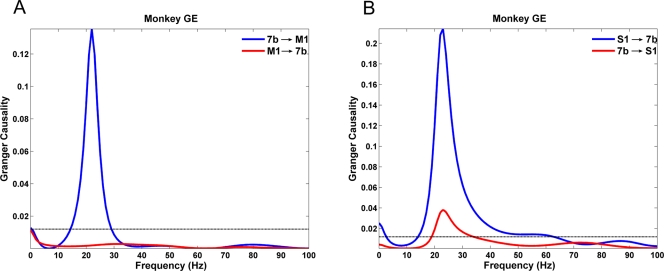
Granger causality spectra. (A) a pair of sites experiencing unidirectional interaction and (B) a site pair experiencing bidirectional interaction in the beta frequency band. The threshold level for significance at p<0.005 is overlaid as a flat line.

**Figure 2:**
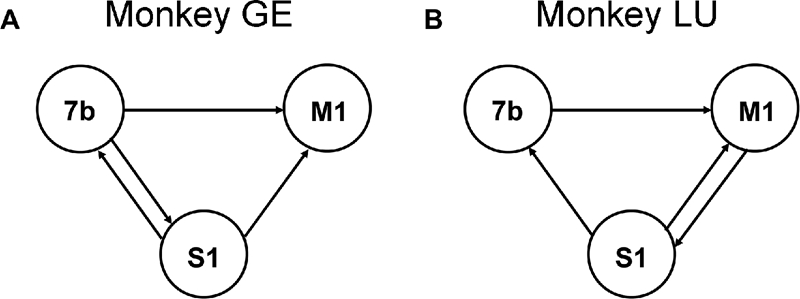
Schematic Granger causality graph. (A) monkey GE and (B) monkey LU. Solid arrows indicate directions of causal influence in the beta frequency band that were significant at p<0.005.

Granger causality spectra are shown in Fig. 1 for a pair of sites experiencing unidirectional interaction (A) and another pair of sites undergoing bidirectional interaction (B). Figure 2 shows the interaction patterns among the three recording sites in both monkeys in the beta frequency range where the same significance threshold criterion described in [11] were used. Except for (S1,7b) in monkey GE and (M1,S1) in monkey LU, the remaining site pairs in both monkeys exhibited unidirectional interaction. Unlike the other five site pairs where the interaction is concentrated in the beta range (see Fig. 1), the (M1,S1) pair in monkey LU also exhibited significant interaction in the gamma frequency range, in addition to that in the beta range. For this pair, the causal influences in the time-domain where instantaneous causality is most readily interpreted, are confounded and is thus excluded from further analysis. Functionally, the observation that S1 and 7b play a pivotal role in the organization of the network has led to the hypothesis that the beta network supports the maintenance of lever depression by facilitating sensorimotor integration [11], [26], [30].

#### Phase-lag distribution

**Figure 3:**
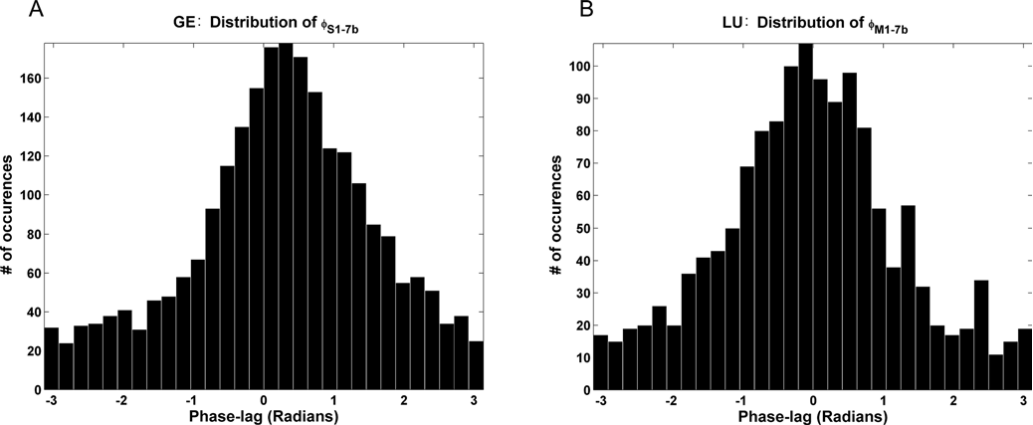
Phase distribution. Histogram of single trial phase-lag values at peak beta frequency for site pairs (A) (S1, 7b) in monkey GE and (B) (M1,7b) in monkey LU.

For a given site pair, the phase-lag at the peak beta frequency was estimated for each trial. Figure 3 shows the phase-lag distributions for two different pairs of sites in monkeys GE and LU. Both distributions are unimodal. In particular, despite a unidirectional interaction pattern between M1 and 7b (Fig. 2(B)), the phase-lag is approximately centered around zero with a mean of 0.04 radians. This suggests that for such pairs the instantaneous causality may contribute significantly to the overall degree of synchrony. Table 3 summarizes the mean phase-lag in the beta band for all five pairs of recording sites.

**Table 3:** Mean phase-lag.

Mean phase-lag in beta band		
	GE	LU
M1,S1	−1.34	-
M1,7b	0.9	0.04
S1,7b	−0.42	−0.4

Mean phase-lags (in radians) measured at the peak frequency in beta band for monkeys GE and LU.

#### Synchrony decomposition and near-zero phase-lag

For recording sites A and B, according to Eqs. (9) and (10), the total synchrony derived from the coherence function can be written as the sum of two directional influences (A→B) and (B→A) and instantaneous causality (A.B). Intuitively, positively correlated common input with no significant relative time delay, measured by the instantaneous causality, has the effect of bringing phase-lag closer to zero. This is particularly so for pairs experiencing unidirectional causal influence. In monkey LU, the phase-lag between M1 and 7b is near zero (Fig. 2(B) and Fig. 3(B)), and not surprisingly, the instantaneous causality in this case makes up 72% percent of total interdependence, a substantial percentage. Below we tested the idea by carrying out an analysis for the site pairs characterized by unidirectional interaction with the analysis protocol outlined in the Methods section.

#### Instantaneous causality and phase-lag

**Figure 4:**
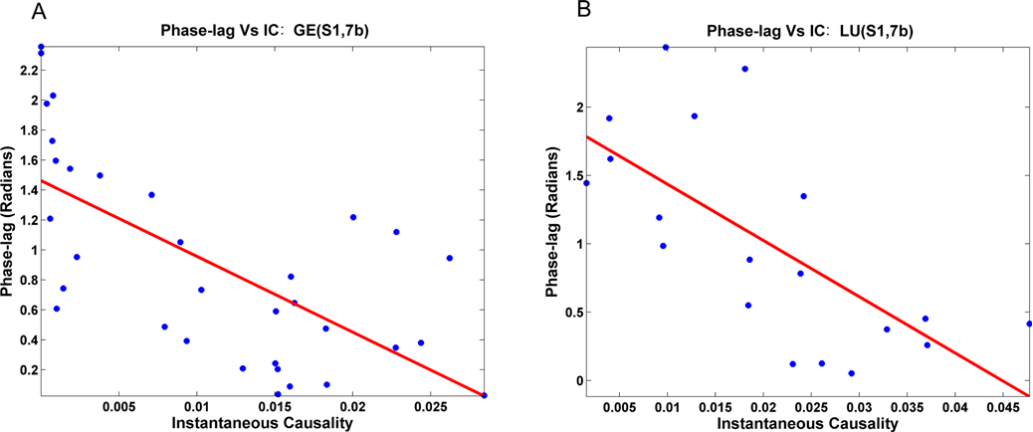
Influence of common input on phase-lag. Scatter plot showing strong negative correlation between instantaneous causality (IC) and magnitude of phase-lag between site pairs (S1,7b) in monkeys (A) GE and (B) LU.

For each site pair the phase-lag was estimated for each trial and the estimated value was used to sort all trials into subensembles. The phase-lag and the instantaneous causality measure for each subensemble constituted a point on a scatter plot. Figure 4 shows the result for (S1,7b) in monkeys GE and LU. Clearly, the two quantities are negatively correlated, indicating that as the instantaneous causality increases, the phase-lag decreases and, in fact, approaches zero. Spearman's rank correlation and Spearman's rank partial correlation coefficients were computed for all the site pairs and listed in Table 4 and 5. All correlation coefficients were negative. Except for (M1,7b) in monkey GE, these correlation coefficients were statistically significant at p = 0.05 level (one tail t-test) (Table 4). By partialing out the effects of the directional influences, the correlation for (M1,7b) also became significant (Table 5).

**Table 4:** Correlation between instantaneous causality and phase-lag.

SRCC between IC & phase-lag		
P<.05	GE	LU
M1,S1	−0.79	-
M1,7b	−0.33 (.058)	−.70
S1,7b	−0.68*	−.73

Spearman's rank correlation coefficient between instantaneous causality and magnitude of phase-lag in the beta band between site pairs for monkeys GE and LU. The results that did not meet significance at p<0.05 are included with their corresponding p-value.

* denotes the channel pair that exhibits bidirectional interaction in the beta frequency band. The symbol “-” indicates that a value is not available.

**Table 5:** Partial correlation between instantaneous causality and phase-lag.

SPRCC between IC & phase-lag		
P<.05	GE	LU
M1,S1	−0.79	-
M1,7b	−0.31	−.44
S1,7b	−0.41*	−.81

Spearman's partial rank correlation coefficient between instantaneous causality and magnitude of phase-lag in the beta band between site pairs for monkeys GE and LU. The partialing is against the directional influences. All the results are significant at p<0.05.

* denotes the channel pair that exhibits bidirectional interaction in the beta frequency band. The symbol “-” indicates that a value is not available.

#### Bidirectional interaction and phase-lag

**Figure 5:**
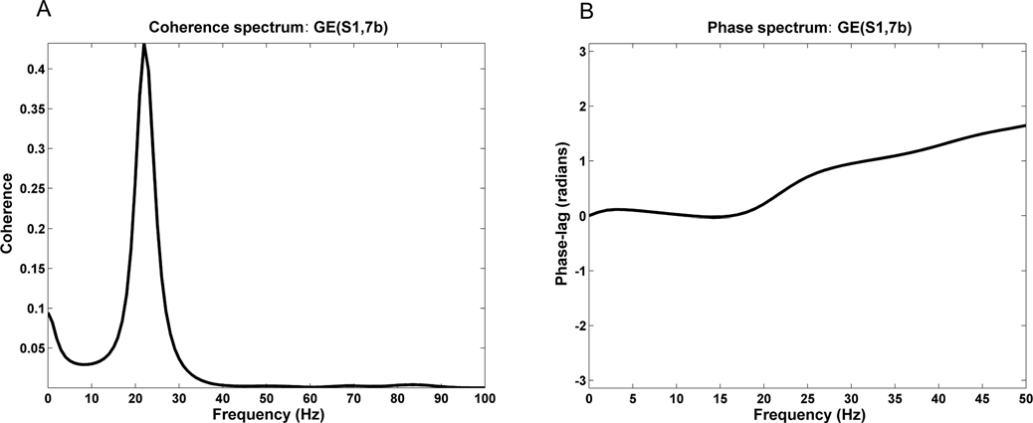
Coherence and phase spectra for site pair (S1,7b) in monkey GE. (A) Coherence spectra for site pair (S1,7b) in monkey GE indicating strong beta band synchrony. (B) The relative phase spectra for the same site pair. The near-zero phase-lag in the beta band is the result of both positively correlated common input and reciprocal interaction.

Inspection of the causality spectrum between the site pair (S1,7b) in monkey GE revealed the presence of bidirectional interaction in the beta band (Fig. 1(B) and Fig. 2(A)). The coherence function and the associated phase spectrum are shown in Figure 5. This channel pair was further analyzed to identify the effect of bidirectional interaction on phase-lag. The strength of directional interaction is expressed as the sum of feedforward and feedback influences. After partialing out the influence of the common input, the magnitude of phase-lag was found to be negatively correlated with the strength of reciprocal interaction (r = −0.455, p = 0.0045). This result supports our early assertion that, in addition to instantaneous causality, bidirectional interaction may also contribute to near-zero phase-lag.

## Discussion

The relative phase between two neural signals A and B at a given frequency, referred to as phase-lag here, can be calculated from the cross-spectrum. For decades, the sign and magnitude of phase-lag have been used to infer direction of information transmission and delay [32]. Increasingly, phase-lag is found to be near-zero in synchronous cortical networks, sometimes involving distant sites. Such phenomenon renders the use of phase-lag as a measure to identify directional influences ineffective. Recently, the phenomenon of near-zero phase-lag has been examined from the point of view of neuronal communication and is considered a manifestation of the brain integrating information from diverse sources [1], [8], [11], [16]. Two factors, among possibly others, could be identified that contribute to the formation of near-zero phase-lag: (a) positively correlated common input with no significant relative time delay and (b) bidirectional interaction. Reports of near-zero phase-lag arising in networks with a predominantly unidirectional interaction pattern further highlights the importance of the first factor.

The influence of the two factors on phase-lag was tested on simulated datasets generated by bivariate autoregressive models. First, it was observed that, for a linear system with unidirectional interaction, a near-zero phase-lag was unlikely in the absence of an instantaneously positively correlated common input. As such an input is introduced and increased, phase-lag is seen to decrease and approach zero. A similar influence of instantaneously positively correlated common input on phase-lag was also observed in networks with bidirectionally interacting. Second, for the case of a bidirectionally interacting system with no common input, increase in the strength of both feed-forward and feed-back interaction leads to the reduction in the magnitude of phase-lag. It is worth noting that not all reciprocally interacting systems exhibit near-zero phase-lag synchrony. The actual phase-lag in a network is likely a function of such factors as relative delays involved in the feed-forward and feed-back pathways and the strength/type of coupling.

The empirical testing of the above ideas faces considerable challenge as a standard correlation or coherence analysis do not offer sufficient information on the relation between the two signals A and B. Recently, advanced connectivity tools have been proposed [25]–[28], [32]–[40] which aim at parsing the synchrony into directional interaction. Mathematically, a theorem by Geweke promises deeper insights [27]. It states that the total interdependence between A and B can be written as the sum of three contributing factors: (A→B), (B→A) and (A.B). The arrow is understood in the sense of Granger causality and (A.B) signifies instantaneous causality which could be interpreted as reflecting the effect of a common input. In the present study Geweke's theorem was applied to study the contribution of the two factors identified above to near-zero phase-lag.

Local field potentials from primary somatosensory (S1), primary motor (M1), and posterior parietal (7b) areas from two monkeys performing a sensorimotor integration task were analyzed. A beta oscillatory network involving all three sites was identified by coherence. The total interdependence between two sites was then decomposed into its directional components. Out of five distinct pairs of recording sites studied, four exhibited predominantly unidirectional interaction in the beta band. The phase-lag was near-zero for one of the five pairs and relatively small for another three. Unlike simulated models, neither the strength of input correlation nor the strength of feed-forward/feed-back interaction can be manipulated to infer their influences on phase-lag. The sorting of trials according to their phase-lag is a strategy to deal with this problem. By sorting the trials according to single trial estimated phase-lag, a negative correlation was found between the phase-lag and instantaneous causality for all pairs of sites, implying that the stronger is the common input the closer to zero is the phase-lag. Despite this tendency, the actual value of the phase-lag for a given pair depends on the relative contribution of the each of the factors in Eqs. (9) and (10), and may vary broadly [from 0.04 (near-zero) to −1.34 (far-from-zero), see Table 3].

If the common input is negatively correlated, then the stronger is this input the closer to ±π is the phase-lag. A careful inspection of the five pairs of recording sites revealed that the noise terms in their respective autoregressive models (see Eq. (1)) were all positively correlated, with the exception of the pair (S1,7b) in GE, where the noise terms was negatively correlated. Since the order of the recording leads used for the bipolar derivation was arbitrary, the signal from S1 was reversed in polarity, which is equivalent to a depth-to-surface subtraction. This correction enables the data from all five pairs to be considered under the same hypothesis. Channel pair (S1, 7b) in monkey GE also has another differing characteristic: the interaction is bidirectional in the beta range. In light of the earlier discussion, the bidirectional interaction in addition to common input could also contribute to the observed near-zero phase-lag. This prediction was confirmed by a partial correlation analysis between the phase-lag and the strength of feedforward and feedback interaction after statistically removing the influence of the instantaneously correlated common input.

In sum, based on our simulated as well as experimental data, for two cortical regions engaged in unidirectional interaction, a positively correlated common input with no significant relative time delay, quantifiable by instantaneous causality, is likely a main contributor tor the near-zero phase-lag between the sites. On the other hand, for two cortical areas engaged in bidirectional interaction, near-zero phase-lag synchrony can emerge as a result of reciprocal interaction or positively correlated common input or a combination of both. Geweke's decomposition theorem, combined with the analysis protocol outlined in the Methods section, can help to ascertain the exact network mechanism for a given problem. Each measure obtained through this decomposition technique has the desirable feature that they all have clear physiological correspondence. For example, bidirectional interaction is highly interpretable in terms of the anatomical connectivity principle of reciprocity in the cortex [41]. Instantaneous causality/common input may be taken to collectively reflect activation of one or several cortical or subcortical regions that project to the sampled sites. Volume conduction, while a possible contributor to instantaneous causality, is unlikely a factor in the present study as bipolar derivation localizes neural activity to its generator. However, for scalp EEG, the influence of volume conduction is known to be significant and must be carefully taken into consideration [42], [43].
